# Markers of anti-malarial drug resistance in *Plasmodium falciparum *isolates from Swaziland: identification of *pfmdr1*-86F in natural parasite isolates

**DOI:** 10.1186/1475-2875-9-68

**Published:** 2010-03-03

**Authors:** Sabelo V Dlamini, Khalid Beshir, Colin J Sutherland

**Affiliations:** 1 Faculty of Health Sciences, University of Swaziland, Mbabane, Swaziland; 2 Department of Infectious and Tropical Diseases, London School of Hygiene and Tropical Medicine, Keppel St, London WC1E 7HT, UK

## Abstract

**Background:**

The development of *Plasmodium falciparum *resistance to chloroquine (CQ) has limited its use in many malaria endemic areas of the world. However, despite recent drug policy changes to adopt the more effective artemisinin-based combination (ACT) in Africa and in the Southern African region, in 2007 Swaziland still relied on CQ as first-line anti-malarial drug.

**Methods:**

Parasite DNA was amplified from *P. falciparum *isolates from Swaziland collected in 1999 (thick smear blood slides) and 2007 (filter paper blood spots). Markers of CQ and sulphadoxine-pyrimethamine (SP) resistance were identified by probe-based qPCR and DNA sequencing.

**Results:**

Retrospective microscopy, confirmed by PCR amplification, found that only six of 252 patients treated for uncomplicated malaria in 2007 carried detectable *P. falciparum*. The *pfcrt *haplotype 72C/73V/74I/75E/76T occurred at a prevalence of 70% (n = 64) in 1999 and 83% (n = 6) in 2007. Prevalence of the *pfmdr1*-86N allele was 24% in 1999 and 67% in 2007. A novel substitution of phenylalanine for asparagine at codon 86 of *pfmdr1 *(N86F) occurred in two of 51 isolates successfully amplified from 1999. The *pfmdr1*-1246Y allele was common in 1999, with a prevalence of 49%, but was absent among isolates collected in 2007. The 86N/184F/1246D *pfmdr1 *haplotype, associated with enhanced parasite survival in patients treated with artemether-lumefantrine, comprised 8% of 1999 isolates, and 67% among 2007 isolates. The *pfdhfr *triple-mutant 16C/51I/59R/108N/164I haplotype associated with pyrimethamine resistance was common in both 1999 (82%, n = 34) and 2007 (50%, n = 6), as was the wild-type 431I/436S/437A/540K/581A/613A haplotype of *pfdhps *(100% and 93% respectively in 1999 and 2007). The quintuple-mutant haplotype *pfdhfr/pfdhps*-CIRNI/ISGEAA, associated with high-level resistance to SP, was rare (9%) among 1999 isolates and absent among 2007 isolates.

**Conclusions:**

The prevalence of *pfcrt *and *pfmdr1 *alleles reported in this study is consistent with a parasite population under sustained CQ drug pressure. The low prevalence of dhps-437G and dhps-540E mutations (ISGEAA) and the rarity of quintuple-mutant haplotype pfdhfr/pfdhps-CIRNI/ISGEAA suggest that SP retains some efficacy in Swaziland. Anti-malarial policy changes in neighbouring countries may have had an impact on the prevalence of molecular markers of anti-malarial resistance in Swaziland, and it is hoped that this new information will add to understanding of the regional anti-malarial resistance map.

## Background

The emergence and spread of drug resistant malaria poses the greatest challenge to the control and elimination of malaria in Swaziland. Despite reports of chloroquine failure suspected to be a result of drug resistance in the 1980s [1], CQ has remained the first-line drug for treatment of uncomplicated *Plasmodium falciparum *malaria in Swaziland. The combination of sulphadoxine and pyrimethamine (SP) has been an effective and affordable alternative single dose treatment for CQ-resistant parasites since its first introduction to Africa in the 1960s. Use of SP increased rapidly in countries with CQ resistance over the next 30 years. As a result, resistance to SP is now globally widespread [2], leading most malaria endemic countries in Africa to abandon SP alone as first-line treatment. In Swaziland, SP was introduced in 1993 as a second-line treatment for uncomplicated malaria following reports of CQ failure in Mozambique [3-5], South Africa (KwaZulu-Natal) [6] and Swaziland [1]. To this day, SP remains a second-line anti-malarial in Swaziland despite its withdrawal as a first-line anti-malarial in KwaZulu-Natal (2001) and Mozambique (2002). Resistance to CQ is associated with polymorphisms in the *pfcrt *and *pfmdr1 *genes, and resistance to SP with *pfdhfr *and *pfdhps *polymorphisms. No data are available concerning the diversity of these loci in Swaziland.

The mutation at codon 76 of the *pfcrt *gene, where lysine is replaced by threonine (K76T) has been strongly associated with *P. falciparum *resistance to CQ and subsequent treatment failure [7]. Mutations in the *pfcrt *gene at codons other than K76T are said to modulate CQ resistance and/or compensate deleterious effects of K76T mutations [8]. Mutations in the *pfmdr1 *gene (N86Y, Y184F, S1034C, N1042D and D1246Y) have been reported to modulate the level of CQ resistance [9-11]. Recent studies have further associated some alleles of the *pfmdr1 *gene with decreased susceptibility to artemisinin-containing drugs. Artemether-lumefantrine (AL) is currently the most favoured combination therapy against uncomplicated *Plasmodium falciparum *malaria in southern Africa. However, *in vivo *studies of AL-treated patients have shown enhanced survival of parasites carrying the *pfmdr1 *polymorphisms (86N, 184F and 1246D) and selection against the YYY haplotype [12,13]. Studies have also reported an increased *pfmdr1 *copy number in parasites of Asian origin following AL treatment [14]. Selection of the NFD haplotype and increased *pfmdr1 *copy number could result in reduced susceptibility of parasites to AL and further investigation of *pfmdr1 *in areas implementing AL treatment policy are clearly needed.

Resistance to SP has been shown to occur following changes in the amino acid sequences of the dihydrofolate reductase (DHFR) [15] and dihydropteroate synthase (DHPS) [16] enzymes, with both *in vitro *and *in vivo *studies finding associations between mutations in the *dhfr *gene of *P. falciparum *and resistance to pyrimethamine [17,18] and between mutations in the *pfdhps *gene and resistance to sulphadoxine [19,20]. The level of resistance to SP has been shown to increase with accumulation of mutations in codons 51, 59, 108 and 164 in *pfdhfr *and in codons 437 and 540 in *pfdhps *[21,22]; the contribution to resistance of mutations at positions 431, 436, 581 and 613 of *pfdhps *remains unclear [23].

This report describes molecular screening of 79 isolates from Swaziland collected in 1999 and 2007. Resistance-associated polymorphisms in the *pfcrt, pfmdr1*, *pfdhfr *and *pfdhps *genes were determined by multiplex qPCR and direct DNA sequencing of conventional PCR products.

## Methods

### Study samples

Two hundred and fifty-two blood samples were collected from patients clinically diagnosed with uncomplicated malaria in eight health centres in the malaria endemic lowveld of Swaziland between 01 February and 30 April, 2007. Only six of these patients were confirmed microscopy positive for *P. falciparum *by two laboratory technicians working independently. Twenty-four of the microscopy-negative samples (10%) were randomly selected to further confirm the validity of the laboratory confirmation by PCR. Seventy-three glass microscope slides with Giemsa-stained thick blood smear samples from confirmed malaria cases in 1999 were also obtained for the analysis. These came from Simunye, on the northern lowveld of Swaziland and Big Bend in the Ubombo Ranches on the southern lowveld of Swaziland. The two centres attract patients from the whole malarious region of Swaziland, with Simunye providing diagnosis and treatment for patients from the northern part and Big Bend for patients from the south.

The study was approved by the London School of Hygiene and Tropical Medicine Ethics Committee and the Ministry of Health and Social Welfare (Swaziland) Ethics Committee. Written informed consent was obtained from all participants or parents/guardians in the case of children.

### Isolation of DNA

Parasite genomic DNA was extracted from dried filter papers using the Chelex^®^100 method according to methods described elsewhere [24]. Briefly, the discs were lysed in 5% saponin in 1 × PBS and incubated at 37°C overnight. The samples were centrifuged, saponin and debris were removed using a vacuum pump, and the pellets washed twice in buffered saline. The samples were then suspended in 6% Chelex^®^100 resin and heat-sealed in deep 96-well plates. The samples were incubated in boiling water for 20 - 25 minutes and then centrifuged to remove resin. Approximately 100 μl of supernatant containing DNA was removed and stored at -20°C.

Parasite DNA was isolated from stained thick blood smears using the QIAmp Mini Blood Purification kit (QIAGEN, UK) following the manufacturer's instructions. A sterile scalpel was used to spread lysis buffer over the blood and to scrape material into a 1.5 ml microcentrifuge tube for further processing. The DNA was finally collected in 100 μl of elution buffer, centrifuged at 8 000 g for 1 min and stored at -20°C.

### Parasite genotyping by PCR

#### pfcrt

The genotype of the *pfcrt *gene for polymorphisms C72S, M74I, N75E, and K76T was determined in at least two multiplex real-time qPCR runs with full agreement using the Rotorgene 3000 platform (Corbett Research, Australia). Primer sets and cycling conditions were as described previously [25], in the presence of the double-labelled probes representing CVIET, CVMNK and SVMNT haplotypes. 3D7, Dd2 and 7G8 DNA obtained from the Malaria Research & Reference Reagent Resource (MR4, Manassas, Vermont, USA) was used to provide sequence-specific positive controls and nuclease-free water was included as a negative control.

#### pfmdr1

Amplification of the *pfmdr1 *gene was initially performed in two fragments (FR1 and FR2). Primers and cycling conditions used for FR1 and FR2 are described elsewhere [13]. For some samples, FR2 amplification was unsuccessful, and so two smaller fragments, FR3 and FR4, encompassing the codons of interest in FR2, were amplified. Primers and cycling conditions for FR3 and FR4 are listed in Table 1.

**Table 1:** *pfmdr1 *PCR primer sequences and reaction conditions used in Fragments 3 and 4 amplification reactions

Gene Fragment	Primer name		Primer Sequence	Codons	PCR Cycling conditions
**Fragment 3**					

Primary FR3	MDRF3N1	F	5'-GCATTTTATAATATGCATACTG-3'	1034, 1042	94°C 3 min/[94°C 30 s-56°C 60 s 65°C 50 s] × 30 cycles 65°C 5 min/15°C 5 min
	MDRF3R1	R	5'-GGATTTCATAAAGTCATCAAC-3'		
Nested FR3	MDRF3N2	F	5'-GGTTTAGAAGATTATTTCTGTA-3'		
	MDRF3R1	R	5'-GCATTTTATAATATGCATACTG-3'		

**Fragment 4**					

Primary FR4	MDRF4N1	F	5'-CAAACCAATCTGGATCTGCAG-3'	1246	94°C 3 min/[94°C 30 s-55°C 60 s-65°C 40 s] × 30 cycles 65°C 5 min/15°C 5 min
	MDRF4R1	R	5'-CAATGTTGCATCTTCTCTTCC-3'		
Nested FR4	MDRF4N2	F	5'-GATCTGCAGAAGATTATACTG-3'		
	MDRF4R1	R	5'-CAATGTTGCATCTTCTCTTCC-3'		

**Sequencing**					

FR3	MDRF3N2	F	5'-GGTTTAGAAGATTATTTCTGTA-3'		96°C 1 min [96°C 30 s-50°C-60°C 4 min] × 26 cycles
	MDRF3R1	R	5'-GCATTTTATAATATGCATACTG-3'		
FR4	MDRF4N2	F	5'-GATCTGCAGAAGATTATACTG-3'		
	MDRF4R1	R	5'-CAATGTTGCATCTTCTCTTCC-3'		

FR - Fragment F - forward R - Reverse

NB: Cycling conditions are the same for primary and nested PCRs.

#### *pfdhfr *and *pfdhps*

Amplification of *pfdhfr *and *pfdhps *genes involved primers and cycling conditions described elsewhere [23,26]. All amplicons of the *pfmdr1, pfdhfr and pfdhps *genes were re-amplified in a nested PCR step. The PCR products of nested reactions were separated by gel electrophoresis on a 1.2% agarose gel stained with ethidium bromide to identify amplified bands of DNA under ultra-violet illumination. Amplicons from nested PCR reactions were purified using the QIAquick PCR Purification Kit (QIAGEN, UK) according to manufacturer's instructions and subjected to di-deoxy fluorescent sequencing (BigDye 3.1, Applied Biosystems, UK) using conditions and sequencing primer pairs described elsewhere [13,25,26] or as shown in Table 1 (*pfmdr1 *FR3 and FR4). The sequence of amplified DNA products was determined using ABI PRISM 3730 Genetic Analyser (Applied Biosystems, UK). Chromas software (Technelysium, Australia) was used to analyse the sequence results. The DNA sequence was compared with reference sequence of the *pfmdr1, pfdhfr *and *pfdhps *portions of the *P. falciparum *3D7 clone using BLAST similarity alignment (Washington University, USA). Appropriate control DNA samples with known *pfmdr1, pfdhfr *and *pfdhps *sequences were used in parallel with field-collected parasite isolates in every step of the protocol.

## Results

### *Pfcrt *polymorphisms

The *pfcrt *72-76 haplotypes CVIET, CVMNK and SVMNT were successfully identified in 70 DNA samples. Five (83%) of the six 2007 samples and 45 (70%) of the sixty-four 1999 samples carried the CVIET haplotype. Only one (17%) of the 2007 samples carried the wild type CVMNK haplotype while it occurred alone in five (8%) of the 64 successfully amplified 1999 samples (Table 2). Mixed haplotype infections (CVMNK/CVIET) were found in 14 (22%) of the 1999 samples. No parasites carried mixed CVIET/CVMNK haplotypes among the 2007 samples. The SVMNT haplotype was not found in any of the isolates. These results suggest that the prevalence of the CVIET haplotype remained high between 1999 and 2007.

**Table 2:** Prevalence of *pfcrt*, *pfmdr1, pfdhfr *and *pfdhps *haplotypes in Swaziland: 1999 and 2007.

Gene	n	Genotype/Haplotype		Prevalence	
				
				1999	2007
***pfcrt*** *Amino acids:* *72 - 76*	64 (1999)	CVMNK	wild-type	5 (8%)	1 (17%)
	6 (2007)	CVIET	mutant	45 (70%)	5 (83%)
		CVMNK/CVIET	mixed	14 (22%)	0 (0%)

***pfmdr1*** *Amino acids:* *86,184,103, 1042, 1246*.	51 (1999)	86N	wild-type	12 (24%)	4 (67%)
	6 (2007)	86**Y**	mutant	37 (73%)	2 (33%)
		86**F**	mutant	2 (4%)	0 (0%)
	50 (1999)	184Y	wild-type	39 (78%)	2 (33%)
	6 (2007)	184**F**	mutant	11 (22%)	4 (67%)
	51 (1999)	1246D	wild-type	25 (51%)	6 (100%)
	6 (2007)	1246**Y**	mutant	25 (49%)	0 (0%)
	
	50 (1999)	NYD	wild-type	2 (4%)	0 (0%)
	6 (2007)	N**F**D	single-mutant 1	4 (8%)	4 (67%)
		**F**YD	single-mutant 2	2 (4%)	0 (0%)
		NY**Y**	single-mutant 3	1 (2%)	0 (0%)
		**Y**YD	single-mutant 4	15(30%)	2 (33%)
		**Y**Y**Y**	double-mutant 1	19 (38%)	0 (0%)
		N**FY**	double-mutant 2	4(8%)	0(0%)
		**YF**D	double-mutant 3	2(4%)	0 (0%)
		**YFY**	triple-mutant	1 (2%)	0 (0%)

***pfdhfr*** *Amino acids:* *50,51,59,108,164*	34 (1999)	CNCSI	wild-type	3 (8%)	1 (17%)
	6 (2007)	CNCSI/C**IRN**I	mixed	0 (0%)	1 (17%)
		C**IRN**I	triple-mutant	28 (82%)	3 (50%)
		C**IRN**I/C**IRNL**	mixed	0 (0%)	1 (17%)
		C**IRN**I/C**I**C**N**I	mixed	3 (10%)	0 (0%)

***Pfdhps*** *Amino acids:* *436, 437, 540, 581,613*.	27 (1999)	SAKAA	wild-type	25 (93%)	6 (100%)
	6 (2007)	S**GE**AA	double-mutant	2 (7%)	0 (0%)

***pfdhr & pfdhps***	23 (1999)	CNCSI/SAKAA		3 (13%)	1 (17%)
	6 (2007)	C**IRN**I/SAKAA		18 (78%)	5(83%)
		C**IRN**I/S**GE**AA	quintuple-mutant	2 (9%)	0 (0%)

NB: C**IRN**I/C**IRNL**, C**IRN**I/C**I**C**N**I and CNCSI/C**IRN**I mixed haplotypes have been grouped into C**IRN**I for analysis of the prevalence of combined *dhfr/dhps *mutant haplotypes. n = number of successfully genotyped samples. Mutant alleles are in bold for ease of identification.

### *Pfmdr1 *polymorphisms

Fifty samples from 1999 were successfully genotyped for *pfmdr1 *at codons 86, 184, 1034, 1042 and 1246, as were all 2007 samples. No mutations were found in codons 1034 and 1042 in any isolates. Therefore, analysis was concentrated on codons 86, 184 and 1246 of *pfmdr1*. Prevalence of the wild-type *pfmdr1*-86N allele was 24% (n = 51) in 1999 and 67% in 2007 (n = 6). Unexpectedly, a novel mutation showing substitution of asparagine (N) with phenylalanine (F), rather than the common tyrosine (Y) substitution, at the 86 position was observed in two (4%) of the 1999 isolates from Swaziland. This mutation has not been described in natural field isolates before, but has been observed in two mefloquine-selected sub-clones of the laboratory isolate W2mef [27]. Its origin or implications are unknown. The mutant 184**F **and 1246**Y **alleles were found in 11/50 (22%) and 25/51 (49%) respectively among 1999 samples. The 184**F **allele showed an increase in prevalence from 22% in 1999 to 67% in 2007. The 1246**Y **allele was not observed among the 2007 isolates.

**Figure 1:**
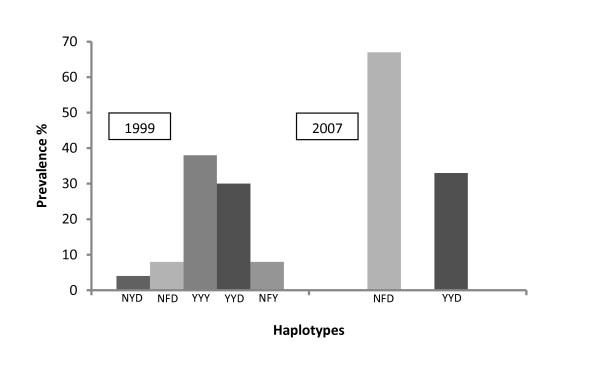
**Prevalence of the five most common *pfmdr1 *haplotypes at codons 84, 184 and 1246 among Swaziland *P. falciparum *isolates in 1999 (N = 50) and 2007 (N = 6)**.

Nine different haplotypes were obtained from the analysis of the 86, 184 and 1246 codons (Table 2). The wild type *pfmdr1-*NYD haplotype was observed in only two (4%) of the 51 successfully sequenced 1999 samples but was not observed in any of the 2007 samples. The prevalence of the single-mutant **Y**YD haplotype remained unchanged in the two years (33% and 30%, respectively). Prevalence of the N**F**D haplotype was only 8% in 1999, but was 67% in 2007. The double-mutant haplotype **Y**Y**Y **was found in many of the 1999 samples (19/50 = 38%) yet in none of the 2007 samples. This was also true of the N**FY **haplotype, which was found in 4/50 (8%) of the 1999 samples but not in the 2007 samples (Figure 1). Other haplotypes occurred with low frequency in 1999 but were absent in 2007 and have not been shown in the Figure. These are **YF**D (4%), NY**Y **(2%), **F**YD (4%) and the triple-mutant haplotype **YFY **(2%).

### *Pfdhfr *polymorphisms

The 2007 isolates (n = 6) were all successfully genotyped for *pfdhfr *mutations at codons 16, 50, 51, 59, 108 and 164, while the DNA samples obtained by scraping thick blood films from 1999 ran low: only 34 of the 73 isolates from 1999 (47%) were successfully genotyped. The wild-type CNCSI haplotype was relatively uncommon, occurring in 8% of isolates in 1999 and 17% of isolates in 2007. Mixed infections, containing the triple-mutant C**IRN**I and double-mutant C**I**C**N**I, were observed in three (10%) of the 1999 isolates. The prevalence of the C**IRN**I triple-mutant haplotype of the *dhfr *gene was 82% (28 of 34) in 1999, and 50% (3 of 6) in 2007, but a statistical test for evidence of a significant reduction is not presented due to the low number of isolates available for the 2007 analysis. Despite reports of the *pfdhfr *I164L mutation in eastern Africa [28], this allele was not found among Swaziland isolates. The prevalence of each *pfdhfr *haplotype identified is shown in Table 2.

### *Pfdhps *polymorphisms

Twenty-seven isolates from 1999 and all six isolates from 2007 were successfully genotyped for *dhps *mutations at the 431, 436, 437, 540, 581 and 613 codons. All the 2007 isolates were wild-type ISAKAA while 93% of the 1999 isolates carried this wild-type haplotype. The remaining 7% carried the double-mutant IS**GE**AA haplotype. The codon 431 mutation (I431V), recently described from Nigerian isolates [23], was not observed.

Twenty-three samples were successfully genotyped for both *dhfr *and *dhps *mutations. The prevalence of the combined wild-type haplotype *dhfr*/*dhps*-CNCSI/SAKAA was similar in 1999 (13%) and 2007 (17%). The triple-mutant haplotype *dhfr*/*dhps*-C**IRN**I/ISAKAA also remained highly prevalent at 78% (1999) and 83% (2007) in the two years. Prevalence of the quintuple-mutation C**IRN**I/IS**GE**AA was 9% (2 of 23) in 1999 and 0% among the isolates collected in 2007. Other mutations in *dhfr *including S108**T **and A16**V **were not found in any of the isolates from Swaziland described herein.

## Discussion

Malaria is still a major concern in Swaziland despite recent reports of significant reduction of disease incidence by more than 95% [29]. Selection of effective and affordable anti-malarials for treatment and control remain extremely important and complicated in this era of emergence of *P. falciparum *parasites resistant to multiple anti-malarials. CQ failure in Swaziland was first reported in 1987 [1]. More than twenty years later, Swaziland retained CQ as a first-line anti-malarial for treating uncomplicated malaria. Although SP has not been used as a first-line anti-malarial in Swaziland, it is still of interest to determine whether parasites remain susceptible to the drug especially following reports of resistance from neighbouring countries. SP is used as a second-line anti-malarial for CQ resistant (CQR) parasites.

The high prevalence of the *pfcrt *CVIET haplotype, which originated in CQR parasites from Southeast Asia [30,31], and the absence of CQR haplotype SVMNT (South American) among isolates examined in this study is consistent with the earlier presumed spread of CQR parasites from Southeast Asia to Africa via the Indian subcontinent. Reports of SVMNT occurrence described in isolated areas of East Africa are probably a result of resistance to AQ or its metabolite desethyl-amodiaquine (DEAQ) following use of AQ as monotherapy [32]. High prevalence of 76T mutations both in 1999 and 2007 suggests that CQ resistance is widespread in Swaziland. Studies have also shown that acquisition of 76**T **mutations favours severity and multiplicity of malaria infection [33] and, together with *pfmdr1 *mutations, is associated with life-threatening complications such as severe anaemia (Hb<5 g/dl) in young children [34].

No published data are available on the *in vivo *efficacy of CQ in Swaziland. Evidence of heavy over-prescription is described here, in which only six of 252 patients treated for malaria in 2007 proved to be parasite positive by both retrospective slide-reading and PCR. Previous studies elsewhere have shown that the 76**T **and 86**Y **mutations occur among parasites surviving CQ treatment, including those responsible for production of transmissible gametocytes [35,36]. In CQ-treated children, parasites with *pfmdr1 *86**Y **and *pfcrt *76**T **are transmitted more frequently than other genotypes and this mechanism probably supports continued circulation of these genotypes [37]. Further studies among malaria patients in Swaziland are required to estimate any impact of these mutations on treatment outcome. The *pfmdr1*-1246**Y **allele, found at 49% prevalence among 1999 isolates, was absent among 2007 isolates, suggesting withdrawal of CQ use may lead to further beneficial changes in the parasite population in Swaziland.

The novel 86**F **mutation in *pfmdr1 *identified in two samples from this study has not previously been described in field-collected parasites. In the isolates described here, the phenylalanine at position 86 is encoded by the codon UUU, which has two base changes from the wild-type asparagine codon AAU, but only a single base-change from the common CQ-resistance-associated tyrosine codon, UAU. The 86**F **allele is therefore likely to have arisen from the 86**Y **form of *pfmdr1 *by a single point mutation, rather than directly from the wild-type 86N form, which would require a minimum of two changes. This is consistent with *in vitro *observations [27].

The absence of the wild-type *pfmdr1*-NYD haplotype in 2007 and its low prevalence among 1999 isolates suggest that CQ resistance had developed over a long time. The high prevalence of the *pfmdr1*-N**F**D haplotype in 2007 and absence of the **Y**YY haplotype is of interest, as studies elsewhere in Africa have reported selection for the N**F**D allele of *pfmdr1 *and loss of the **Y**Y**Y **allele following treatment with artemether-lumefantrine (AL) [12,13]. Since there is no history of AL use in Swaziland, this pattern may be a result of the impact of drug policy changes in neighbouring South Africa, KwaZulu-Natal (KZN) and Mozambique. Swaziland is a very small country and the influence of drug policies in Mozambique and South Africa (KZN) cannot be ruled out. KZN introduced AL in 2001 while Mozambique introduced artesunate-sulphadoxine/pyrimethamine (AS-SP) combination therapy in 2002 and artesunate/AQ in 2004. Thus, deployment of AL in KZN and the introduction of AS-SP treatment policy in Mozambique could have influenced selection of 86N parasites through successful transmission compared to their mutant counter-parts. However, this phenomenon can only be confirmed through analysis of a larger number of samples from Swaziland. Transmission studies reported a four-fold reduction in transmissibility to mosquitoes of the 86Y allele compared to the 86N allele following addition of artesunate to CQ [35]. The same study reported an association between CQ monotherapy and increased 86**Y **transmissibility. Other studies have shown increased tolerance of laboratory parasite clones to mefloquine and halofantrine, (which are related to lumefantrine) if they carried the 86N allele [10] or the 1034S/1042N/1246D haplotype [38]. Therefore, the prevalence of N**F**D haplotypes in Swaziland has to be closely monitored as it could compromise the effectiveness of combinations involving lumefantrine in the future. Unfortunately, data on the prevalence of N**F**D and **Y**Y**Y **haplotypes in KZN following AL implementation in 2001 are not available.

Regional drug pressure, as well as prescription of SP to CQ-resistant cases probably partly explains the high prevalence of *dhfr *triple-mutant haplotypes (82%) observed in 1999 in Swaziland. The prevalence of the *dhfr*-C**IRN**I triple-mutant haplotype in Zone 1 in Southern Mozambique, the zone closest to Swaziland, was 47.5% in 1999 [39] which is lower than that observed in Swaziland, suggesting only partial influence from Mozambique, possibly because SP was in use in Swaziland as a second-line drug. In contrast, there was a high prevalence of the triple-mutant haplotype at Masvold Hospital in northern KZN [40] in 1999. The results given here suggest that the prevalence of *dhfr *mutations in Swaziland was high compared to that in neighbouring countries. Thus, regional drug pressure alone may not explain allele prevalence in Swaziland. SP drug pressure in Swaziland may also have been further enhanced by the use of other anti-folate compounds, such as the combination antibiotic cotrimoxazole, which have cross-resistance with SP. As HIV infection rates are high in Swaziland, cotrimoxazole is commonly prescribed as prophylaxis against opportunistic infections to HIV patients [2], and may have contributed to persistence of C**IRN**I triple-mutant malaria parasites in Swaziland.

The results presented here strongly support the recent move to replace CQ with the highly efficacious artemisinin-combination therapies (ACTs) in Swaziland. The replacement of presumptive treatment with a quick, simple and cheaper diagnostic method such as rapid diagnostic tests (RDTs) to reduce inappropriate prescription of drugs should reduce the cost of ACT drug and further delay development of resistance. Deployment of AL in Swaziland is likely to further reduce prevalence of **Y**Y**Y **haplotype but could increase the N**F**D haplotype, and this may help preserve good AQ efficacy [12,13,41], as will the recent withdrawal of AQ use in neighbouring Mozambique. In the absence of the *pfmdr1 *YYY haplotype, AQ shows *in vivo *and *vitro *efficacy against parasite clones with the CVIET *pfcrt *haplotype [13,42]; parasites of this genotype are shown here to be abundant in Swaziland.

## Conclusion

This study is the first to document the presence and circulation of CQR parasites in Swaziland. The high prevalence of *pfcrt*-76T, *pfmdr1*-86Y, and *pfmdr1*-1246Y among 1999 isolates suggests that the population of isolates circulating in Swaziland has been selected by the long use of CQ. These findings thus strongly support the withdrawal of CQ as a first-line anti-malarial drug for uncomplicated *P. falciparum *malaria, and replacement with ACT. SP remains available for use in pregnant women, but the occurrence of the S**GE**AA haplotype in Swaziland, previously associated with resistance to sulphadoxine, suggests that SP use for treatment of uncomplicated malaria might rapidly select for these SP-resistant parasites. A weakness of this study is the small number of isolates obtained during the health facility survey in 2007. Genetic analysis of parasites from larger surveys in Swaziland and neighbouring countries is required for better understanding of the development of anti-malarial drug resistance in this region.

## Competing interests

The authors declare that they have no competing interests.

## Authors' contributions

SD and CJS conceived the study. SD performed fieldwork. SD and KB performed laboratory work. SD and CJS analysed data. All authors contributed to the paper, and approved the final manuscript.
